# Impact of an interprofessional shared decision-making and goal-setting decision aid for patients with diabetes on decisional conflict – study protocol for a randomized controlled trial

**DOI:** 10.1186/s13063-015-0797-8

**Published:** 2015-06-27

**Authors:** Catherine H. Yu, Noah M. Ivers, Dawn Stacey, Jeremy Rezmovitz, Deanna Telner, Kevin Thorpe, Susan Hall, Marc Settino, David M. Kaplan, Michael Coons, Sumeet Sodhi, Joanna Sale, Sharon E. Straus

**Affiliations:** Li Ka Shing Knowledge Institute of St. Michael’s Hospital, 30 Bond Street, Toronto, ON M5B 1W8 Canada; Department of Medicine, University of Toronto, Toronto, ON Canada; Dalla Lana School of Public Health, University of Toronto, 30 Bond Street, Toronto, ON M5B 1W8 Canada; Department of Family and Community Medicine, University of Toronto, Toronto, ON Canada; Family Practice Health Centre, Women’s College Hospital, University of Toronto, 76 Grenville Street, Toronto, ON M5S 1B3 Canada; Institute for Health System Solutions and Virtual Care, Women’s College Hospital, University of Toronto, 76 Grenville Street, Toronto, ON M5S 1B3 Canada; School of Nursing, University of Ottawa, Ottawa, ON Canada; Clinical Epidemiology Program, Ottawa Hospital Research Institute, 451 Smyth Road, Ottawa, ON K1H 8M5 Canada; Department of Family and Community Medicine, University of Toronto, Sunnybrook Hospital, Room 101, 2075 Bayview Avenue, Toronto, ON M4N 3M5 Canada; Southeast Toronto Family Health Team, Toronto East General Hospital, 833 Coxwell Avenue, East York, ON M4C 3E8 Canada; North York Family Health Team, 703-240 Duncan Mill Road, Toronto, ON M3B 3S6 Canada; Department of Psychology, York University, 4700 Keele Street, Toronto, ON M3J 1P3 Canada; Department of Family and Community Medicine, Toronto Western Hospital, University Health Network, Toronto, ON, Canada; Mobility Program Clinical Research Unit, Li Ka Shing Knowledge Institute, St. Michael’s Hospital, 30 Bond Street, Toronto, ON M5B 1W8 Canada

**Keywords:** Cluster randomized controlled trial, Diabetes mellitus, Interprofessional care, Medical informatics, Patient education, Pilot study, Priority setting, Patient decision aid, Qualitative methods, Shared decision-making, Study protocol

## Abstract

**Background:**

Competing health concerns present real obstacles to people living with diabetes and other chronic diseases as well as to their primary care providers. Guideline implementation interventions rarely acknowledge this, leaving both patients and providers feeling overwhelmed by the volume of recommended actions. Interprofessional (IP) shared decision-making (SDM) with the use of decision aids may help to set treatment priorities. We developed an evidence-based SDM intervention for patients with diabetes and other conditions that was framed by the IP-SDM model and followed a user-centered approach. Our objective in the present study is to pilot an IP-SDM and goal-setting toolkit following the Knowledge-to-Action Framework to assess (1) intervention fidelity and the feasibility of conducting a larger trial and (2) impact on decisional conflict, diabetes distress, health-related quality of life and patient assessment of chronic illness care.

**Methods/Design:**

A two-step, parallel-group, clustered randomized controlled trial (RCT) will be conducted, with the primary goal being to assess intervention fidelity and the feasibility of conducting a larger RCT. The first step is a provider-directed implementation only; the second (after a 6-month delay) involves both provider- and patient-directed implementation. Half of the clusters will be assigned to receive the IP-SDM toolkit, and the other will be assigned to be mailed a diabetes guidelines summary. Individual interviews with patients, their family members and health care providers will be conducted upon trial completion to explore toolkit use. A secondary purpose of this trial is to gather estimates of the toolkit’s impact on decisional conflict. Secondary outcomes include diabetes distress, quality of life and chronic illness care, which will be assessed on the basis of patient-completed questionnaires of validated scales at baseline and at 6 and 12 months. Multilevel hierarchical regression models will be used to account for the clustered nature of the data.

**Discussion:**

An individualized approach to patients with multiple chronic conditions using SDM and goal setting is a desirable strategy for achieving guideline-concordant treatment in a patient-centered fashion. Our pilot trial will provide insights regarding strategies for the routine implementation of such interventions in clinical practice, and it will offer an assessment of the impact of this approach.

**Trial registration:**

Clinicaltrials.gov Identifier: NCT02379078. Date of Registration: 11 February 2015.

**Electronic supplementary material:**

The online version of this article (doi:10.1186/s13063-015-0797-8) contains supplementary material, which is available to authorized users.

## Background

Competing patient and physician priorities and competing disease priorities present challenges in the provision of care for individuals with complex medical needs and multiple comorbidities [1]. Shared decision-making (SDM) tools, or decision aids, can be used to help prioritize treatment options and have the potential to improve patient care. The authors of a systematic review of randomized controlled trials (RCTs) of SDM identified 11 studies [2], 2 of which examined long-term decisions that occurred over multiple sessions in a 9- and 12-month time frame and reported improved adherence, reduced depression and improved well-being.

Despite the potential for SDM to improve patient-centered outcomes, implementation of SDM in care has been limited because of clinician factors (such as limited time, lack of applicability to the patient situation [3]) and patient factors (such as patient–provider power imbalance, health literacy and denial about the condition [4]). However, facilitators of SDM include provider motivation to engage in SDM, the perception of having a positive impact on the clinical process and outcome [5], health care provider training, patient-mediated interventions [6], provision of medical knowledge, validation of patient experiences, strong interpersonal skills and provider availability [4].

The use of an interprofessional (IP) team approach may further facilitate the process of SDM. Diabetes care occurs in the context of IP care [7], which has been shown to improve clinical outcomes [8–10]. Furthermore, IP care may facilitate uptake of SDM [11]. Hence, an IP approach to SDM is the process whereby two or more health care professionals are involved in making the decision with the patient [12]. This approach may occur synchronously, but it more often occurs asynchronously and therefore requires a shared framework with a common understanding. Achieving a common understanding of the essential elements of the SDM process among the IP team, as well as recognizing the influence of the various individuals on this process, will improve success in reaching a shared decision.

SDM can be facilitated by the use of patient decision aids (PtDAs) [6, 13, 14], as they help frame the decision to be made. The authors of a 2014 Cochrane review of PtDAs identified 115 studies and found that PtDAs improved decision quality and process and that, when used within the consultation with the health care practitioner, patients were more likely to achieve SDM with their practitioner compared with patients using a PtDA on their own [6, 15]. We identified six PtDAs focused on diabetes, one evaluated with a prospective observational study [16], four evaluated through RCTs [15, 17–22] and one RCT protocol [23]. These PtDAs included a goal-setting intervention [16, 22], Diabetes Medication Choice [17], Statin Choice [15, 18, 19, 21] and a metabolic control aid [20]. These were studied in specialty clinics [15, 16, 18, 19, 23], in primary care settings [17, 21, 22] and among the general public [20]. Whereas knowledge, risk perception, satisfaction, decision-making participation, trust, decisional conflict and documented goals improved, there was no impact on diabetes empowerment or clinical outcomes. The effect on medication adherence was mixed. In one before-after study and one RCT, researchers have examined the impact of goal-setting decision aids [16, 22]. In the first study [16], the intervention (consisting of a 28-page patient workbook, a 2-hour patient education session and two 2-hour medical resident seminars) was delivered in a primary care clinic and resulted in increased knowledge (*P* = 0.001), number of documented diabetes goals (pre: 0.67 goals; post: 1.09 goals; *P*< 0.001), but no improvement in glycemic control, weight or diabetes empowerment score were noted. In the second study, the investigators examined the impact of another goal-setting and SDM aid in 344 patients with uncomplicated type 2 diabetes in 18 Dutch primary care practices [22]. This electronic medical record–generated decision aid presented to patients their individually calculated risks of vascular disease based on the patients’ cardiometabolic profile. No difference was found in patient empowerment for setting and achieving goals. These interventions did not specifically address patient-important priorities and preferences. In addition, effectiveness may be optimized by providing a point-of-care tool for use at the time of consultation and a provider-specific tool, emphasizing longitudinal use, and more explicitly integrating the IP team.

We hypothesize that a multicomponent PtDA toolkit (patient-directed, provider-directed and point-of-care tools) that helps to individualize care priorities and incorporates an IP approach to SDM may help to implement complex guideline recommendations for patients with type 1 or type 2 diabetes and other comorbidities and may also improve the decision-making process and quality as well as patient-centered outcomes. We elected to examine this population, given the added relevance of a goal-setting aid in patients with multiple competing health care concerns. The overall goal of this study is to assess intervention fidelity (i.e., if, how and when the toolkit is actually used in clinical care) as well as the feasibility of scaling up the intervention and study to a larger RCT to determine the efficacy and effectiveness of the SDM tool to improve clinical outcomes.

## Methods/Design

### Study overview

In this protocol, we describe the fifth and sixth phases of a multiphase program of research described elsewhere [1]. We adopted the Knowledge-to-Action Framework and engaged knowledge users throughout the process by including patients, primary care providers and other stakeholders as members of the research team to optimize implementation [24]. Briefly, this program consists of six phases, with the first four being feasibility testing (phase 1), IP-SDM toolkit development (phase 2), heuristic evaluation (phase 3) and usability testing (phase 4). Throughout the development process, the toolkit will be refined iteratively based on findings of each phase. We will then conduct an evaluation of the effectiveness of the PtDA toolkit by conducting a two-step, pilot clustered RCT (phase 5), followed by individual interviews (phase 6). We used the SPIRIT checklist to report this protocol [25].

The initial aim of this phase is to assess intervention fidelity and the feasibility of conducting a larger cluster RCT to evaluate efficacy and effectiveness of the SDM tool on clinical outcomes. The subsequent aim is to evaluate the impact of the toolkit on a variety of patient-reported outcomes, including patient decisional conflict, diabetes distress, health-related quality of life and chronic care delivery.

### Phases 5 and 6: cluster randomized controlled trial and individual interviews

#### Study design and objectives

An exploratory, two-step, parallel-group, clustered RCT with a 1:1 allocation ratio will be conducted. We chose to use a cluster RCT design to minimize contamination of the control group and avoid biased estimates of effect size [26].

The first step will be provider-directed (the toolkit will be delivered only to the providers, including physicians, nurses, dietitians and/or pharmacists); the second step (which will occur 6 months later) will be provider- and patient-directed (the toolkit will also be delivered to the patient). The use of the two-step approach will allow us to monitor uptake by population (provider or patient) as well as barriers influencing uptake in our study setting. Although patient-directed interventions improve health behaviors and outcomes [27], optimal delivery of PtDA (provider- or patient-directed strategy) is unclear. When mailed to patients, viewing rates were as low as 25 %, whereas strategies dependent on providers failed, as the providers did not see this as their role and were distracted by competing demands [28]. We selected a 6-month interval for each step to ensure sufficient time for health care providers and patients to follow up, as well as to enable adequate exposure to use of the PtDA to influence our study outcomes.

#### Phase 5: cluster randomized controlled trial

##### Setting and participants

Family practice groups will be recruited from nine local health integration networks located in southern Ontario, Canada. These networks were designated by the Ontario Ministry of Health and Long-Term Care to plan, integrate and fund local health services, such as health service providers, community care access centers, community health centers, community support services, hospitals and long-term care facilities [29]. We will exclude groups that do not have an IP staff that includes a nurse, dietitian or pharmacist or that do not have an electronic medical record system capable of identifying patients with diabetes. The research team will contact medical directors of eligible practices to explain the project and solicit their participation. Two to five physicians from each family practice group will be invited to participate.

Using electronic medical records at each consenting physician’s practice, the research coordinator will identify patients with diabetes and two other comorbidities [heart disease (including ischemic, valvular, congestive, arrhythmic and congenital disease), stroke, hypertension, cancer survivor (excluding nonmelanoma skin cancer), chronic lung disease, arthritis, inflammatory bowel disorders and urinary incontinence]. Those patients who do not speak English, have documented cognitive deficits, are unable to give informed consent, have limited life expectancy (<1 year) or are not available for follow-up will be excluded. In addition, patients who are involved in concurrent diabetes-related initiatives or who are seen by resident physicians will be excluded, as this does not represent usual clinical care. Patients who are seen by resident physicians will be excluded because of limited involvement of the IP team, smaller patient practices and limited resident availability owing to other training needs.

##### Intervention arm

PtDA development has been described previously [1]. We developed an IP-SDM toolkit based on the IP-SDM framework [30] and according to the International Patient Decision Aids Standards criteria [31]. The IP-SDM framework consists of seven steps in SDM and considers the role of the patient, the family and the health care team. These steps are (1) assessment of decision to be made, (2) information exchange, (3) values and preferences, (4) feasibility, (5) preferred choice, (6) actual choice and (7) implementation [12, 32]. Actions involved in our toolkit include obtaining the patient’s cardiometabolic and psychosocial profile, determining the patient’s general priorities for care, eliciting the patient’s diabetes-specific goals and outcomes, outlining diabetes-specific therapies and their benefits and risks and synthesizing these items and the patient’s selected strategies into an action plan. The tool ranks goals and strategies based on the evidence underlying the clinical practice guidelines (CPG) recommendation as well as patient values. Use of the IP-SDM toolkit within the team will be contextualized to each site and based on the usual roles, responsibilities and processes of care of the health care team. The IP-SDM toolkit is in English, and the patient-directed components target a grade 8 literacy level (i.e., readability score) [31].

At study start, the toolkit, consisting of the online decision aid, a one-page provider enabler (a laminated sheet summarizing the purpose and flow of the decision aid with step-by-step instructions and accompanying screenshots), a similar one-page patient enabler and brief training videos for both providers and patients will be distributed by the research coordinator to each of the health care providers in practices randomized to the toolkit; this constitutes the provider-directed intervention phase (step 1). After 6 months, a link to the PtDA will be emailed to eligible patients; this constitutes the provider- and patient-directed phase (step 2). On the basis of the final results of phases 1–4 (ongoing at time of protocol publication), we will incorporate other strategies to overcome barriers to use and optimize implementation before trial initiation.

##### Control arm

At study start, a paper copy of the executive summary of the Canadian Diabetes Association (CDA) clinical practice guidelines and a postcard outlining online clinical information resources will be distributed to each of the health care providers in practices randomized to the control group. After 6 months, copies of a CDA patient education pamphlet regarding diabetes self-management and a postcard outlining online additional patient resources will be mailed to eligible patients. These provider- and patient-directed guideline dissemination tools (not incorporating SDM) have been launched widely and are also publicly accessible on the CDA guidelines website (guidelines.diabetes.ca).

##### Outcome measures

*Initial aim*: The feasibility of conducting a larger cluster RCT to improve diabetes-related clinical outcomes will be assessed by recruitment and retention of study participants through evaluating study procedures and processes, including recruitment response rate and number of eligible patients, time required to recruit adequate sample size, questionnaire response rate, willingness of participants to be randomized and willingness of clinicians to recruit patients. We will deem the study feasible if we are able to complete recruitment within a 12-month period and attain a questionnaire response rate of 75 % or higher for completion of at least 75 % of the patient questionnaires. This will inform whether we will proceed with a larger trial or if we need to modify study processes before going forward. Intervention fidelity will be assessed by patterns of use of the intervention through examination of website use statistics, such as frequency and duration of specific components of the intervention and patterns of use over time.

*Subsequent aim*: The primary outcome is decisional conflict [33]. Secondary outcomes are diabetes distress [34], health-related quality of life [35], chronic illness care [36] and intention to engage in IP-SDM [37]. These outcomes will be assessed by using well-validated, patient-completed questionnaires at baseline, 6 months and 12 months through online surveys (Table 1). These questionnaires take approximately 15–20 minutes to complete. These outcomes were selected because they are direct measures of knowledge use by patients that will allow us to better understand mediating variables of knowledge use, such as patient activation, goal-setting, problem-solving and decision-making support. Decisional conflict was chosen to allow us to assess the impact of our PtDA on the quality of the decision-making process, an important first measure of the effectiveness of a PtDA [38] and the SDM process [39]. Diabetes distress was selected as a holistic and patient-centered measure of knowledge use that uniquely acknowledges patient prioritization of health care goals [40, 41]. Health-related quality of life was chosen to complement diabetes-specific quality of life to assess general health, given the nature of the PtDA and complex patient population selected [42]. Patient perception of chronic illness care was selected to assess key elements of the chronic care model namely, patient activation, goal-setting, problem-solving and/or contextual counseling, delivery system design and/or decision-making support and follow-up and/or coordination [36]. Providers’ intention to engage in SDM will be assessed with an 11-item questionnaire based on the theory of planned behavior [37]. Surrogate clinical outcomes (e.g., hemoglobin A1C level) were not chosen, owing to their limited relevance to the individualized nature of our toolkit.

**Table 1:** Schedule of enrollment, interventions and assessments

	Study period					
	Enrollment	Allocation	Postallocation			Closeout
Time point	−12 mo	0	0	6 mo	12 mo	16 mo
Enrollment						
Eligibility screen	X					
Informed consent	X					
Allocation		X				
Intervention arm						
Provider-mediated phase						
Patient-mediated phase						
Control arm						
Provider-mediated phase						
Patient-mediated phase						
Assessments						
Baseline variables	X					
Providers: age, sex, duration in practice, practice load, remuneration plan, academic or community practice, rural or urban practice, solo or group practice						
Patients: age, sex, ethnicity, age at diagnosis, comorbidities, smoking status, educational attainment, annual income						
Outcome variables						
Logs of all study processes to assess recruitment and retention						
Website use statistics					X	
Decisional conflict, diabetes distress, health-related quality of life, chronic illness care, intention to engage in IP-SDM			X	X	X	
Qualitative interviews						X

*Abbreviation: IP-SDM* interprofessional shared decision-making

##### Sample size calculation

On the basis of a previous study in which the decisional conflict scale [43] was used, a clinically meaningful effect size of 0.4 with a standard deviation of 0.6, α of 0.05 and β of 0.10 will be employed for sample size calculations. With at least 40 patients per physician, a 50 % participation rate and an anticipated attrition rate of 25 %, we expect about 15 patients per site to participate. Previous data have shown that the ρ-value (intraclass correlation coefficient) is 0.013 for decisional conflict for patients with diabetes clustered within primary care physicians [44]. Therefore, the sample size required, accounting for clustering, is 56 patients per intervention and control group, or 4 sites per intervention and control group.

##### Randomization

Practices will be simultaneously randomized and allocated by a biostatistician to either the intervention or control arm using computer-generated randomization in a 1:1 ratio. A biostatistician will take the list of all eligible patients from each cluster and generate by computer a random order list. Assuming a 50 % response rate to recruitment, we will select the first 40 patients from this random order list and invite them to participate. If we are not able to recruit 20 patients from this initial group of 40, we will send out additional invitations to the subsequent participants from this random order list.

##### Risk of bias

After assignment, investigators, research coordinators or trial participants will no longer be blinded to group allocation, owing to the nature of the intervention. Each practice will be given a code, and the biostatistician will analyze the data blindly. Codes will be accessed after analysis is completed.

##### Data collection

*Initial aim*: The research team will keep detailed records prospectively of recruitment methods employed, using a log for each level of recruitment, comprising family practice groups, physicians and patients (Additional file 1). To assess intervention fidelity, we will collect website use statistics by analyzing server logs upon study completion (Additional file 2).

*Subsequent aim*: At trial entry, sociodemographic information will be obtained (for providers: age, sex, duration in practice, practice load, remuneration plan, academic or community practice, rural or urban practice, solo or group practice; for patients: age, sex, ethnicity, age at diagnosis, comorbidities, smoking status, educational attainment, annual income). Data on outcomes will be collected by using participant-completed questionnaires (Additional file 3) submitted (either online or mailed according to patient preference) at 6-month intervals and at study completion, corresponding to intervention steps 1 and 2 for a total of 3 data points over a 12-month period. Participants will be reminded twice by email or telephone at 2-week intervals to complete the questionnaires. Data will be stored on the secure, password-protected institutional server. All hard-copy data will be stored in a locked and secured filing cabinet at the research institute.

##### Analysis

*Initial aim:* We will conduct basic frequency and descriptive statistical analysis of our retention, recruitment and use objectives for intervention fidelity and feasibility.

*Subsequent aim:* We hypothesize that patients in the intervention arm will have reduced decisional conflict. We hypothesize they will have reduced diabetes distress and improved quality of life and chronic care delivery. Analysis will be done by intention to treat. The analysis to test this hypothesis will be carried out using multilevel hierarchical models (generalized estimating equations for binary outcomes and linear mixed-effects models for continuous outcomes) to account for the clustered nature of the data. The group assignment will be a physician-level variable. The robustness of the primary analysis to missing data will be assessed by imputing best and worst case scenarios for missing data. We will also assess the impact of sociodemographic variables on these outcomes, as the literature demonstrates that not all patients and providers want SDM [45–47]. In addition, to facilitate better planning for a subsequent larger trial, standard deviations of our primary outcome measures will be used to more precisely estimate sample size for the future.

##### Data monitoring

We did not create a data monitoring committee, given the pilot nature of this study, low risk to trial participants and assessment of less serious outcomes. We will keep a prospective log of adverse events and other unintended effects.

#### Phase 6: individual interviews

##### Participants

Individual interviews will be conducted with patients, their family members and their health care providers following completion of the trial. We will use purposive sampling to recruit participants with a range of experiences and characteristics [48] from among the broader pool of trial participants who were randomized to the intervention arm. Fifteen to twenty interviews in total are anticipated to reach theoretical saturation [48].

##### Data collection

To assess intervention fidelity, we will assess toolkit use and the manner with which it was used (e.g., which components, which individuals). To assess the feasibility of scaling up to a larger cluster RCT, we will evaluate the acceptability of the toolkit and intervention, reasons for use and nonuse, and barriers to and facilitators of use.

We will use a semistructured interview guide to elicit participants’ views regarding the IP-SDM process and intervention use. The details will include acceptability, usability, strengths and weaknesses of the intervention; facilitators of and barriers to its use, user satisfaction; and sustainability of its use (Additional file 4). We will make the intervention available during each interview.

##### Data analysis

All interviews will be audiotaped and transcribed verbatim [49]. Transcripts will be analyzed inductively using a constant comparative approach to identify emergent themes [50]. Coding will be conducted independently by two team members with expertise in qualitative research methods. The coding framework will be developed and then iteratively tested and refined with subsequent interviews [50]. NVivo software (version 9; QSR International, Doncaster, Australiawill be used to assist with data management and retrieval.

### Research ethics

The study was approved by the research ethics boards (REBs) of St. Michael’s Hospital (reference number 13-014C), Sunnybrook Health Sciences Health Center (reference number 345-2013), Women’s College Hospital (reference number 2013-0058), Toronto East General Hospital (reference number 609-1410-Mis-245), North York General Hospital (reference number 13-0265), Southlake Regional Health Center (reference number 0055-1314) and Markham-Stouffville Hospital. Protocol amendments will be submitted to the relevant REB before trial participants are informed, and they will be registered at ClinicalTrials.gov. Eligible patients will be mailed an information letter (Additional file 5), then called the following week. If the patients are interested in participating in the study, they will be asked to complete an online consent form. We will obtain informed consent from each participant. To protect confidentiality, study identification numbers will be used on study surveys. The master log that links study identification numbers to any personal information will be kept separately. Research team members will have access to the final trial dataset. We do not anticipate any harm to be caused by trial participation.

### Dissemination

Trial results will be communicated to study participants by letter, to each study site by presentation and to academic audiences through publications and conference presentations. International Committee of Medical Journal Editors authorship guidelines will be followed.

## Discussion

Diabetes is a complex chronic disease, and its management is further complicated by other common comorbidities. Adopting a patient-centered SDM approach whereby patients, supported by their health care team, set goals and management priorities has the potential to improve the delivery of care and patient-important outcomes, such as quality of life, as well as the uptake of patient-relevant guideline recommendations. Our objective is to pilot and evaluate a diabetes-focused SDM and priority-setting PtDA in IP primary care practices.

Effective implementation of SDM and PtDAs into clinical practice requires practical delivery mechanisms [51, 52]. We will identify barriers to and employ facilitators of implementation identified in the literature [3–6] and in earlier phases of this project. We will also assess intervention fidelity and use in our feasibility study and interviews.

The study’s strengths include its randomized design and clustered nature, use of validated patient-important outcomes, knowledge-user engagement (primary care providers, people with diabetes), varied team expertise (SDM, knowledge translation, information technology, primary care diabetes, and qualitative and quantitative research methods), dual coding of interview transcripts and triangulation of qualitative and quantitative results [48, 50, 53].

The limitations and challenges of this study potentially include volunteer bias and inadequate recruitment. We will assess volunteer bias by comparing sociodemographic characteristics of participants and nonparticipants, which will further inform recruitment in a subsequent, larger, definitive trial. We hope to optimize successful recruitment and implementation with our combined knowledge user-researcher team.

IP-SDM has not been taken up extensively in clinical care, but it has the capacity to help focus the care of patients with multiple comorbidities in the face of an overwhelming volume of recommendations. By assessing intervention use and fidelity, barriers to and facilitators of implementation, and patient-important outcomes, this study will contribute to the understanding of its role in clinical care in our evolving, complex population, and it has the potential to impact chronic care delivery and outcomes that are of relevance to patients with multiple comorbidities.

## Trial status

Recruitment of study sites and participants is ongoing

## Additional files

Additional file 1:
**Recruitment log.**
Additional file 2:
**Website use statistics.**
Additional file 3:
**Description of primary and secondary outcome scales.**
Additional file 4:
**Semistructured Interview Guide.**
Additional file 5:
**Information and consent form.**

## Abbreviations

CDA: Canadian Diabetes Association
CPG: Clinical practice guidelines
IP: Interprofessional
PtDA: Patient decision aid
REB: Research ethics board
SDM: Shared decision-making
