# Supplementary material for: Impact of an interprofessional shared decision-making and goal-setting decision aid for patients with diabetes on decisional conflict – study protocol for a randomized controlled trial
Source: Trials. 2015 Jun 27;16:286. doi: 10.1186/s13063-015-0797-8 (PMC4486130; doi:10.1186/s13063-015-0797-8)
Supplement: Additional file 1: — Recruitment log. [file 13063_2015_797_MOESM1_ESM.pdf]

Appendix 1: Methods: Recruitment Log Items

Level of recruitment	Stage of recruitment	Data Items
Site recruitment	Enrollment	FHT name<br>Method of contact<br>Email<br># email follow-up<br>Responded<br>Additional information provided<br>Presentation<br>Consented<br>Consented via<br>Reasons declined
Physician recruitment	Enrollment	FHT name<br>Date initiated<br>#eligible physicians<br>Method of contact<br>Email<br># email follow-up<br>Responded<br>Additional information provided<br>Presentation<br>Consented<br>Consented via<br>Reasons declined<br>Date completed

	Allocation	Allocated to<br><br>Baseline questionnaire<br><br>Received intervention<br><br># times tool used<br><br>Did not receive intervention<br><br>Reasons for not receiving
	Follow-up	Complete<br><br>6-month questionnaire completed<br><br>12-month questionnaire completed<br><br>Reasons lost
Patient recruitment	Identification	FHT name<br><br>Date initiated<br><br>Physician name<br><br>Potentially eligible patients<br><br>- Number of rostered patients<br>- Number of patients with diabetes<br>- Number of patients with ≥2 comorbidities<br>Excluded patients<br><br>- Number of patients excluded<br>- Do not speak English<br>- Documented cognitive deficits<br>- Unable to give informed consent<br>- Limited life expectancy (< 1year)<br>- Not available for follow-up<br>- Resident patient
	Random selection	Date completed<br><br># eligible patients (randomized)<br><br># patients contacted
	Enrollment	Date mailed<br><br>Consent

		Consented via<br><br>Reason declined<br><br>Date phoned<br><br>Result of phone call<br><br>Link to consent sent on
	Allocation	Allocated to<br><br>Baseline questionnaire<br><br>Received intervention<br><br># times tool used<br><br>Did not receive intervention<br><br>Reasons for not receiving
	Follow-up	Complete<br><br>6-month questionnaire completed<br><br>12-month questionnaire completed<br><br>Reasons lost
	Analysis	Included<br><br>Excluded<br><br>Reasons why
