# Supplementary material for: Impact of an interprofessional shared decision-making and goal-setting decision aid for patients with diabetes on decisional conflict – study protocol for a randomized controlled trial
Source: Trials. 2015 Jun 27;16:286. doi: 10.1186/s13063-015-0797-8 (PMC4486130; doi:10.1186/s13063-015-0797-8)
Supplement: Additional file 2: — Website use statistics. [file 13063_2015_797_MOESM2_ESM.pdf]

Appendix 2: Methods: Website usage statistics

**Category**	**Item**
User type	Patient<br>Family member<br>Health care provider
Number of times used over study period	Total<br>By user type
Frequency of use over study period	Total<br>By user type
Most frequently revisited pages (by returnees)	By user type
Mean duration of use	Total<br>By page<br>By user type
Median duration of use	Total<br>By page<br>By user type
