# Supplementary material for: Impact of an interprofessional shared decision-making and goal-setting decision aid for patients with diabetes on decisional conflict – study protocol for a randomized controlled trial
Source: Trials. 2015 Jun 27;16:286. doi: 10.1186/s13063-015-0797-8 (PMC4486130; doi:10.1186/s13063-015-0797-8)
Supplement: Additional file 3: — Description of primary and secondary outcome scales. [file 13063_2015_797_MOESM3_ESM.pdf]

Appendix 3: Description of primary and secondary outcome scales (page 1/1)

Outcome	Scale	Description
Decisional conflict	Decisional Conflict Scale (traditional statement format)(32)	This scale consists of 16 items with 5 response categories (0-Strongly Agree, 4-Strongly Disagree), where higher scores indicate greater decisional conflict. The scale includes subscales for uncertainty, informed, values clarity, support and effective decision. Test-retest correlation and Cronbach alpha exceed 0.78. It correlated to related constructs of knowledge, regret and discontinuance, and had excellent predictive validity. A clinically significant effect size is 0.30 to 0.40; Scores lower than 25 are associated with implementing decision; scores exceeding 37.5 are associated with decision delay or feeling unsure about implementation. The primary outcome decisional conflict has been demonstrated to be responsive to change over time and thus will yield meaningful results when measured at baseline and throughout the study intervention.
Diabetes-related quality of life	Diabetes Distress Scale(33)	The DDS is a 17 item instrument that assesses emotional distress and functioning specific to living with diabetes. Responses are scored on a 6-point Likert-type scale from 1 = "no problem" to 6 = "serious problem". Scores can range from 17-102 with higher scores indicating poorer diabetes-related quality of life and lower scores indicating better diabetes-related quality of life. This instrument has been found to have high internal reliability with a Cronbach's alpha of 0.93 , good convergent validity with the Center for Epidemiological Studies Depression Scale (CESD) (r = 0.56) and self-care behaviors including lower adherence to eating recommendations (r = 0.30) and lower levels of physical activity (r = 0.20)
Health-related quality of life	Short Form 36 Health Survey (SF-36)(34)	The SF-36is a widely-used and validated generic measure of health-related quality-of-life. It is a 36-item multidimensional measure of perceived health, assessing physical functioning, physical role, bodily pain, general health, vitality, social functioning, emotional role and mental health. Scores ranges from 0-100, with higher scores reflecting better health. Its validity was demonstrated in studies of diabetic patients(53), and discriminates among patients with comorbidities(54-56)
Chronic care delivery	Patient Assessment of Chronic Illness Care (PACIC)(35)	The PACIC assesses the degree to which care is congruent with the Chronic Care Model from the perspective of the patient. Specifically, it was designed to measure patient activation, goal-setting, problem-solving/contextual counseling, delivery system design/decision support and follow-up/coordination. The PACIC has been used to evaluate a variety of chronic health conditions, including type 2 diabetes (35, 57, 58). It has moderate test-retest reliability (r=0.58 during the course of 3 months) and correlates moderately to measures of primary care and patient activation (r = 0.32– 0.60, median = 0.50, P <0.001).
Intention to engage in SDM	Theory of planned behavior intention questionnaire (36)	This 11-item questionnaire is based on the Theory of Planned Behavior, encompassing instrumental attitude, affective attitude, subjective norm and perceived behavioral control. It has a reliability that ranges from .67 to 0.93(59).
