# Supplementary material for: Impact of an interprofessional shared decision-making and goal-setting decision aid for patients with diabetes on decisional conflict – study protocol for a randomized controlled trial
Source: Trials. 2015 Jun 27;16:286. doi: 10.1186/s13063-015-0797-8 (PMC4486130; doi:10.1186/s13063-015-0797-8)
Supplement: Additional file 4: — Semistructured Interview Guide. [file 13063_2015_797_MOESM4_ESM.pdf]

Appendix 4a: Methods: Semi-structured interview guide (patient and family member)

Thank you for taking the time to meet with me today. My name is_____________ and I work with Dr. Catherine Yu from St. Michael's Hospital (*and other docs from Sunnybrook etc).* We are conducting interviews with people with diabetes, and their family members, who participated in our study using our tool "My Diabetes Plan". We are also interviewing the health care providers who used the tool.

Specifically we will discuss whether you used it, how you used it, why you used it (or not), and what prevented you and made it easier for you to use. The goal of this research project is to increase the involvement of patients in decisions about their health care. Findings from these interviews will help us find out whether and how we were able to help patients like you prioritize their health care goals and make decisions about their diabetes.

The interview should take about 45 to 60 minutes. I will be taping the interview so that I don't miss any of your comments. All of your comments will remain confidential. You don't have to respond to any question that makes you uncomfortable.

Have you had a chance to read the consent form? Do you have any questions about the consent or about what I have just explained to you? Are you willing to participate?

Great, let's start.

[Tool - use]

1. Do you remember using this website? Does this look familiar? [have website open]If so, what do you remember about it?

2. [If used it] Tell me about how you used or continue to use the "My Diabetes Plan"
    a. [Prompts:] with a health care provider, by yourself, or both;
        i. If you used it with a health care provider, which ones?
            1. When did you use it? What prompted you to use it?
        ii. If you used it by yourself, where did you use it? [at home, waiting room] Did anyone at home help you with it? If so, who?
            1. When did you use it? What prompted you to use it?
    b. Why did you use it? What was your motivation for using the site?
    c. How many times did you use it?
        i. [If more than 1 time] What kept you returning to the site? Tell me about that. Why did you go back to using it?
        ii. [If only one time, or stopped using] If you visited the site only once, what kept you from visiting it again?
        iii. What would have encouraged you to visit the site more often?

[Tool – acceptability of use]

3. We would like to know what you felt about this website. What did you think of the website?

[Prompts:]

a. What has your experience with this website been, overall?
b. I have the website open on this computer – if you remember, please show me which pages you used on the website and tell me what you liked and disliked about each of these pages.
c. What content was particularly helpful (or not helpful)?
d. Sometimes too much information can be overwhelming. What did you find with this website? What did you think about the information e.g. was there too much, too little?
e. Do you think the tool addressed both the positive and negative aspects of X? Explain.
f. Did it help you express values that are important to you? How did it do this?
g. Did it help you talk through your diabetes care with your health care provider? How did it do this?
h. How easy was it or not easy, to use the tool? [usability]
i. How clear or not clear did you find the tool? [content]

[Tool - non-use]

4. [If didn't remember using it] We are doing this study to find out if people would use it. Recognizing that all of us have many demands on our attention and time, what do you feel prevented you from accessing the site more often? (prompts: didn't know about it? Competing "life" issues? Competing "health" issues? No interest? No time? Not useful? Better sites?)

[Tool - facilitators and barriers to use]

5. Do you remember using this? Does this look familiar? [have patient 1-page handout]
6. Do you remember watching this? Does this look familiar? [have patient video open and play start]
7. How would encouragement from your physician or other health care providers to use the site have affected how often you visited this site?
8. How would allowing family members or friends to access to the site have an impact on your use of the site?
9. What else would make it easier for you to use this tool?

10. What made it hard for you to use this tool?

[Tool - Sustainability of Use]

11. You have used this decision-making aid for several months. What would keep you coming back in the future?
    a. What did you do when you received an email from the website about going back to the website to review your diabetes goals?
    b. What would you tell other people with diabetes about this website?

[Shared Decision-making and Goal-setting]

12. In the last 1 year, how have decisions or treatment plans about your diabetes been made? (e.g. decisions are made together with a doctor or nurse or dietitian, the doctor or nurse or dietitian makes the decision, the doctor or nurse or dietitian offers options and allows you to make the decision)
    a. How important is it for you to participate in making decisions about your diabetes care? Can you tell me more about that?
    b. What did you think about the way these decisions were made?
    c. How comfortable were you with the way these decisions were made?

13. In the last 1 year, have you set any goals about your diabetes care? For example, you might have said "I'd really like to stay active and be able to be out and about". How have you set goals about your diabetes care? (e.g. decisions are made together with a doctor or nurse or dietitian, the doctor or nurse or dietitian makes the decision, the doctor or nurse or dietitian offers options and allows you to make the decision) [review patient's goals/strategies prior to interview and refer to these]
    a. How important is it for you to set goals about your diabetes care? Can you tell me more about that?
    b. What did you think about the way these goals were set?
    c. How comfortable were you with the way goals were set?

[Interprofessional approach]

14. In the last 1 year, who has helped you manage your diabetes?
    a. How important was it for you to have different kinds of people and health care providers help you manage your diabetes? What are the pros and cons of this approach for you? Explain.
    b. How comfortable were you with having different health care providers help you manage your diabetes?

[Multimorbidity]

15. You have diabetes and at least two additional chronic conditions. This tool addressed only one of your conditions, diabetes.
    - How did it help or not help with you which treatments to start with, given that you have these other chronic conditions?
    - People with diabetes and other chronic disease have many different behavior/lifestyle changes to make. What behavior/lifestyle modifications have you made and how did you decide to make these changes? Did you have to choose one behavior over another depending on one condition being more important than another? Can you give us an example?

Appendix 4b: Methods: Semi-structured interview guide (health care provider)

Thank you for taking the time to meet with me today. My name is_____________ and I work with Dr. Catherine Yu from St. Michael's Hospital (*and other docs from Sunnybrook etc).* We are conducting interviews with health care providers who used the tool, who participated in our study using our tool "My Diabetes Plan". We are also interviewing the people with diabetes, and his/her family members.

Specifically we will discuss whether you used it, how you used it, why you used it (or not), and what prevented you and made it easier for you to use. The goal of this research project is to increase the involvement of patients in decisions about their health care. Findings from these interviews will help us find out whether and how we were able to help your patients prioritize their health care goals and make decisions about their diabetes.

The interview should take about 45 to 60 minutes. I will be taping the interview so that I don't miss any of your comments. All of your comments will remain confidential. You don't have to respond to any question that makes you uncomfortable.

Have you had a chance to read the consent form? Do you have any questions about the consent or about what I have just explained to you? Are you willing to participate?

Great, let's start.

[Tool - use]

16. Do you remember using this decision-making aid? Does this look familiar? [havedecision-making aid open] If so, what do you remember about it?

17. [If used it] When did you use it? How did you use it?
    a. Why did you use it? What was your motivation for using the site?
    b. How many times did you use it?
        i. [If more than 1 time] What kept you returning to the site? Tell me about that. Why did you go back to using it?
        ii. [if only one time, or stopped using] If you visited the site only once, what kept you from visiting it again?
        iii. What would have made you visit the site more often?

[Tool – acceptability of use]

18. We would like to know what you felt were the strengths/weaknesses of this decision-making aid.
    a. What has your experience with this decision-making tool been, overall?
    b. I have the decision-making aid open on this computer – please show me which pages you used on the decision-making aid and tell me what you liked and disliked about each of these pages.
    c. What content was particularly helpful (or not helpful)?

d. Sometimes too much information can be overwhelming. What did you find with this decision-making aid? Was there too much information that was overwhelming? Or, was there enough information to be useful to you as you interacted with the patient?
e. Do you think the tool provided a balanced or unbalanced view of risks and benefits, pros and cons?
f. Did it help your patient express values that were important to him or her? How did it do this?
g. Did it help you talk through your patient's diabetes care? How did it do this?
h. Can you comment on the ease of use of the tool? (e.g. how clear or not clear was it?)

[Tool - non-use]

19. [If didn't remember using it] We are doing this study to find out if people would use it. Recognizing that all of us have many demands on our attention and time, what do you feel prevented you from accessing the site more often? (prompts: Competing "life" issues? Competing "health" issues? No interest? No time? Not useful? Better sites?)

[Tool - facilitators and barriers to use]

20. Do you remember using this? Does this look familiar? [have provider 1-page handout]
21. Do you remember watching this? Does this look familiar? [have provider video open and play start]
22. How would encouragement from your patient, family members, or other health care providers to use the site have affected how often you visited the site?
23. If patients and their family members requested that you access the site, what effect would that have on your use of the site?
24. What else would make it easier for you to use this tool?

25. What made it hard for you to use this tool?

[Tool - Sustainability of Use]

26. You have used the decision-making aid for several months. What would keep you coming back in the future?
    a. What would you tell other people with diabetes about this decision-making aid?

[Shared Decision-making and Goal-setting]

27. In the last 1 year, how have health care decisions about your patient's diabetes been made? (e.g. decisions are made along with another health care professionals? another health care professional makes the decision? you make that decision? You or other health care professionals offer options and allows the patient to make the decision)
    a. How important is it for you that your patient participates in making decisions about his or her diabetes care? What are the pros and cons of this approach for you?
    b. What did you think about the way these decisions were made?

c. How comfortable were you with the way these decisions were made?

28. In the last 1 year, how have you helped your patient set goals about his/her diabetes care? (e.g. decisions are made along with another health care professionals? another health care professional makes the decision? you make that decision? You or other health care professionals offer options and allows the patient to make the decision)[review patient's goals/strategies prior to interview and refer to these]
    a. How important is it for you to help you patients to set goals about his/her diabetes care? What are the pros and cons of setting goals?
    b. What did you think about the way these goals were set?
    c. How comfortable were you with the way goals were set?

[Interprofessional approach]

29. In the last 1 year, who has helped your patient manage his/her diabetes?
    a. How important was it to have different kinds of people and health care providers help manage his/her diabetes? What are the pros and cons of this approach for you?
    b. What did you think about this approach to helping your patient manage his/her diabetes?
    c. How comfortable were you with having different health care providers help your patient manage his/her diabetes?

[Multimorbidity]

30. Many of your patients have diabetes and at least two additional chronic conditions. This tool addressed diabetes and was designed to make diabetes management more focused.
    - How did it help or not help with you which treatments to start with, given that many of your patients had other chronic conditions?
