# Supplementary material for: Impact of an interprofessional shared decision-making and goal-setting decision aid for patients with diabetes on decisional conflict – study protocol for a randomized controlled trial
Source: Trials. 2015 Jun 27;16:286. doi: 10.1186/s13063-015-0797-8 (PMC4486130; doi:10.1186/s13063-015-0797-8)
Supplement: Additional file 5: — Information and consent form. [file 13063_2015_797_MOESM5_ESM.pdf]

Appendix 5a: Methods: Patient information and consent form

(*THIS CONSENT WILL BE ADMINISTERED ELECTRONICALLY, THROUGH A WEBPAGE OR IN PERSON)

Information about a Research Study

Development, testing & implementation of a decision aid for patients with diabetes in clinical care

Purpose and Background of the Research

You have been invited to consider participation in a research study that involves developing and using a diabetes-focused shared decision-making (SDM) and goal-setting decision aid.

Diabetes is a complex disease, often associated with multiple other conditions. Competing disease priorities and competing patient-physician priorities present inter-related challenges in the provision of care for the complex patient, thus reducing guideline adherence. Adopting a patient-centered shared decision-making (SDM) model of care, wherein the patient sets goals and management priorities with the support of health care providers, has the potential to improve uptake of patient-relevant guideline recommendations, improve the process of care and patient-important outcomes such as quality of life.

We need your help in developing this resource so that it is effective in clinical care.

Description of the Research

There are 3 substudies in this project:

> **Substudy 1**: Assessment of the feasibility of an interprofessional SDM tool for use in goal-setting in clinical practice and will develop potential content for this tool.
>
> **Substudy 2:** Evaluation the SDM tool developed after Substudy 1 through usability sessions.
>
> **Substudy 3:** Implementation and evaluation of the SDM in a clinical setting.

You are being asked to participate in **Substudy 3**. You are being invited because you are a health care professional treating people who have diabetes and other related health conditions.

If you agree to participate you will be provided with information designed to enhance your clinical care of patients with diabetes and other related conditions, paired with related patient resources. There are 2 sets of resources that we will be comparing; you will be randomly selected to receive 1 of these 2 sets of resources. You will be asked to use this information in your practice for 9 months. After 9 months, all consenting eligible patients within your practice (patients with diabetes and 2 other chronic comorbidities) will be mailed the related patient resources designed to assist them in managing their diabetes along with other related health conditions. Your patient may bring these resources to his/her appointment with you to discuss it with you. These patients will be asked to use these materials for a 9-month period and will answer a questionnaire regarding their comfort with decision-making related to their diabetes care on 3 occasions during the study period.

You will also be asked to complete a short questionnaire that provides sociodemographic information about yourself.

Study outcomes will be measured by patient-completed questionnaires.

Potential Harms (Injury, Discomforts or Inconvenience):

There are no known harms associated with participation in this study. There is a time requirement for reviewing the information designed to enhance your clinical care of patients with diabetes. This time requirement may present an inconvenience.

Potential Benefits

You may directly benefit as a result of your participation in this study through the opportunity to learn about shared decision making and goal setting for use in your clinical practice. In addition, the investigators hope that this study will help diabetes patients to set goals and management priorities with the support of their health care providers which could improve their quality of life.

Confidentiality and Privacy

Confidentiality will be respected and no information that discloses your identity will be released or published without consent unless required by law. All identifying information will be destroyed. This means that no information will be released or printed that would disclose personal identity. Your identity will not be included in the conduct, data storage, analysis and presentation of findings (data will be presented in aggregate form only). To ensure confidentiality, all identifying features will be removed to protect participant anonymity.

Only the Principal Investigator will have access to the consent forms which will be kept in a locked cabinet for a period of 6 years after the study has been published, then the consent forms will be shredded and disposed of in a secure manner.

You will be assigned a unique study ID that will be recorded in a log accessible only to the study coordinator. All responses to the background questionnaire will be identified only by your study ID so that your responses remain completely confidential. Once all study data has been collected this log will be destroyed.

Costs/ Reimbursement

There is no cost for participation in this study. Although we cannot reimburse you directly for your time, we hope that your participation will improve the care offered to patients with diabetes seen in your practice.

Participation and Withdrawal:

Participation in research is voluntary. Participation or non-participation will not affect your current or future employment. If you choose to participate in this study you can withdraw from the study at any time without any consequences.

Research Ethics Board Contract:

If you have any questions as a research subject you may contact Dr. Bob Hyland, Chair Research Ethics Board at 416 864 6060 ext 2557.

Study Contact

The investigators understand that you may ask questions any time about this study. If you have any questions regarding this study, you should contact Catherine Yu, St. Michael's Hospital, at 416-864-6060 x77532.

(*IF CONSENT IS ADMINISTERED THROUGH WEBPAGE, THIS FORM WOULD BE USED)

<u>Consent:</u>

<u>Study Title:</u>“Development, testing & implementation of a decision aid for patients with diabetes in clinical care”

I acknowledge that the research study described above has been explained to me and that any questions that I have asked have been answered to my satisfaction. I have been informed of the alternatives to participation in this study, including the right not to participate and the right to withdraw. Participation or non-participation will not affect my current or future employment. As well, the potential risk, harms and discomforts have been explained to me and I also understand the benefits (if any) of participating in the research study.

I understand that I have not waived my legal rights nor released the investigators, sponsors, or involved institutions from their legal and professional duties. I know that I may ask now, or in the future, any questions I have about the study or the research procedures. I have been assured that records relating to me will be kept confidential and that no information will be released or printed that would disclose personal identity without my permission unless required by law. I have been given sufficient time to read and understand the above information.

Please click on the “I Agree” or “I Disagree” button below.

(Once participant has clicked on “I agree” the following will pop up)

Thank you for agreeing to participate in the study.

Please provide the following information:

Name:

Telephone contact #:

Email address:

Your consent will be submitted electronically. The study coordinator will contact you soon.

(*IF CONSENT IS ADMINISTERED IN PERSON, THIS FORM WOULD BE USED)

Consent:

Study Title:“Development, testing & implementation of a decision aid for patients with diabetes in clinical care”

I acknowledge that the research study described above has been explained to me and that any questions that I have asked have been answered to my satisfaction. I have been informed of the alternatives to participation in this study, including the right not to participate and the right to withdraw. Participation or non-participation will not affect my current or future employment. As well, the potential risk, harms and discomforts have been explained to me and I also understand the benefits (if any) of participating in the research study.

I understand that I have not waived my legal rights nor released the investigators, sponsors, or involved institutions from their legal and professional duties. I know that I may ask now, or in the future, any questions I have about the study or the research procedures. I have been assured that records relating to me will be kept confidential and that no information will be released or printed that would disclose personal identity without my permission unless required by law. I have been given sufficient time to read and understand the above information.

I hereby consent to participate in this study and will be given a signed copy of this consent form.

________________________ ________________________ _____________

Name of Participant (Print) Signature of Participant Date & Time

I have had a discussion with the participant regarding the study to confirm that the participant understands the nature of the study.

________________________ _________________________ ____________

Name & Position of Person Obtaining Consent (Print) Signature of Person Obtaining Consent Date & Time

Appendix 5b: Methods: Health care provider information and consent form

(*THIS CONSENT WILL BE ADMINISTERED ELECTRONICALLY, THROUGH A WEBPAGE OR IN PERSON)

Information about a Research Study

Development, testing & implementation of a decision aid for patients with diabetes in clinical care

Purpose and Background of the Research

You have been invited to consider participation in a research study that involves developing and using a diabetes-focused shared decision making and goal-setting decision aid.

Diabetes is a complex disease, often associated with multiple other conditions. Diabetes patients are challenged by competing disease priorities and competing patient-physician priorities often making it difficult for the diabetes patient to follow the guidelines offered by their health care professionals. Adopting a model of care, wherein the patient sets goals and management priorities with the support of health care providers, has the potential to improve the ability of the patient to follow the guidelines provided by their health care professionals, improve communication between the patient and the health care professional and improve important outcomes such as quality of life for the patient.

We need your help in developing this resource so that it is effective in clinical care.

Description of the Research

There are 3 substudies in this project:

> **Substudy 1**: Assessment of the feasibility of an interprofessional shared decision making tool for use in goal-setting in clinical practice and development of content for this tool.
>
> **Substudy 2:** Evaluation of the shared decision making tool developed after Substudy 1 through usability sessions.
>
> **Substudy 3:** Implementation and evaluation of the shared decision making in a clinical setting.

You are being asked to participate in **Substudy 3**. You are being invited because you are a patient with diabetes and other related health conditions.

If you agree to participate, you will answer 3 questionnaires, either online or via mail. The first questionnaire will be completed on the day that you enter the study and then again after 9 months and 18 months. The questionnaire will take approximately “15” minutes to complete and will include background information about yourself, your diabetes-related quality of life, how well your and your health care professionals communicate, conflicts that you may feel when making decisions about your health and your assessment of chronic illness care.

At the 9 month time point you will be mailed some material designed to assist you in making decisions about managing your diabetes along with other medical conditions. You will be randomly given one of two possible sets of materials. You may use and discuss these materials with your physician or other health care practitioners.

Potential Harms (Injury, Discomforts or Inconvenience):

There are no known harms associated with participation in this study.

Potential Benefits

There are no direct benefits to you for participation in the study. The investigators hope that this study will help diabetes patients to set goals and management priorities with the support of their health care providers which could improve their quality of life.

Confidentiality and Privacy

Confidentiality will be respected and no information that discloses your identity will be released or published without consent unless required by law. All identifying information will be destroyed. This means that no information will be released or printed that would disclose personal identity. Your identity will not be included in the conduct, data storage, analysis and presentation of findings (data will be presented in aggregate form only). To ensure confidentiality, all identifying features will be removed to protect participant anonymity.

Only the Principal Investigator will have access to the consent forms which will be kept in a locked cabinet for a period of 6 years after the study has been published, then the consent forms will be shredded and disposed of in a secure manner.

You will be assigned a unique study ID that will be recorded in a log accessible only to the study coordinator. All responses to the questionnaires will be identified only by your study ID so that your responses remain completely confidential. Once study data has been collected this log will be destroyed.

Costs/ Reimbursement

There are no costs to involvement in this study. Although we cannot reimburse you directly for your involvement, we hope that your participation will improve the care offered to patients like yourself with diabetes.

Participation and Withdrawal:

Participation in research is voluntary. Participation or non-participation will not affect your current health care or that of your family. If you choose to participate in this study you can withdraw from the study at any time without any consequences.

Research Ethics Board Contract:

If you have any questions as a research subject you may contact Dr. Bob Hyland, Chair Research Ethics Board at 416 864 6060 ext 2557.

Study Contact

The investigators understand that you may ask questions any time about this study. If you have any questions regarding this study, you should contact Catherine Yu, St. Michael’s Hospital, at 416-864-6060 x77532.

(*IF CONSENT IS ADMINISTERED THROUGH WEBPAGE, THIS FORM WOULD BE USED)

Consent:

Study Title:"Development, testing & implementation of a decision aid for patients with diabetes in clinical care"

I acknowledge that the research study described above has been explained to me and that any questions that I have asked have been answered to my satisfaction. I have been informed of the alternatives to participation in this study, including the right not to participate and the right to withdraw. Participation or non-participation will not affect my current or future employment. As well, the potential risk, harms and discomforts have been explained to me and I also understand the benefits (if any) of participating in the research study.

I understand that I have not waived my legal rights nor released the investigators, sponsors, or involved institutions from their legal and professional duties. I know that I may ask now, or in the future, any questions I have about the study or the research procedures. I have been assured that records relating to me will be kept confidential and that no information will be released or printed that would disclose personal identity without my permission unless required by law. I have been given sufficient time to read and understand the above information.

Please click on the "I Agree" or "I Disagree" button below.

(Once participant has clicked on "I agree" the following will pop up)

Thank you for agreeing to participate in the study.

Please provide the following information:

Name:

Telephone contact #:

Email address:

Your consent will be submitted electronically. The study coordinator will contact you soon.

(*IF CONSENT IS ADMINISTERED IN PERSON, THIS FORM WOULD BE USED)

Consent:

Study Title:“Development, testing & implementation of a decision aid for patients with diabetes in clinical care”

I acknowledge that the research study described above has been explained to me and that any questions that I have asked have been answered to my satisfaction. I have been informed of the alternatives to participation in this study, including the right not to participate and the right to withdraw. Participation or non-participation will not affect my current or future employment. As well, the potential risk, harms and discomforts have been explained to me and I also understand the benefits (if any) of participating in the research study.

I understand that I have not waived my legal rights nor released the investigators, sponsors, or involved institutions from their legal and professional duties. I know that I may ask now, or in the future, any questions I have about the study or the research procedures. I have been assured that records relating to me will be kept confidential and that no information will be released or printed that would disclose personal identity without my permission unless required by law. I have been given sufficient time to read and understand the above information.

I hereby consent to participate in this study and will be given a signed copy of this consent form.

______________________	______________________	____________
Name of Participant (Print)	Signature of Participant	Date & Time

I have had a discussion with the participant regarding the study to confirm that the participant understands the nature of the study.

______________________	______________________	____________
Name & Position of Person Obtaining Consent (Print)	Signature of Person Obtaining Consent	Date & Time
